# Vagus Nerve Stimulation Reduces Neuroinflammation Through Microglia Polarization Regulation to Improve Functional Recovery After Spinal Cord Injury

**DOI:** 10.3389/fnins.2022.813472

**Published:** 2022-04-07

**Authors:** Hui Chen, Zhou Feng, Lingxia Min, Weiwei Deng, Mingliang Tan, Jian Hong, Qiuwen Gong, Dongyun Zhang, Hongliang Liu, Jingming Hou

**Affiliations:** Department of Rehabilitation, Southwest Hospital, Third Military Medical University Army Medical University, Chongqing, China

**Keywords:** spinal cord injury, vagus nerve stimulation, microglial polarization, neuroinflammation, cholinergic anti-inflammatory pathway, alpha 7 nicotinic acetylcholine receptor

## Abstract

**Background:**

Spinal cord injury (SCI) is a devastating disease that lacks effective treatment. Interestingly, recent studies indicated that vagus nerve stimulation (VNS), neuromodulation that is widely used in a variety of central nervous system (CNS) diseases, improved motor function recovery after SCI. But the exact underlying mechanism of how VNS ameliorates SCI is unclear. This study aimed to confirm the efficacy and further explore the potential therapeutic mechanism of VNS in SCI.

**Method:**

A T10 spinal cord compression model was established in adult female Sprague-Dawley rats. Then the stimulation electrode was placed in the left cervical vagus nerve (forming Sham-VNS, VNS, and VNS-MLA groups). Basso-Beattie-Bresnahan (BBB) behavioral scores and Motor evoked potentials (MEPs) analysis were used to detect motor function. A combination of histological and molecular methods was used to clarify the relevant mechanism.

**Results:**

Compared with the Sham-VNS group, the VNS group exhibited better functional recovery, reduced scar formation (both glial and fibrotic scars), tissue damage, and dark neurons, but these beneficial effects of VNS were diminished after alpha 7 nicotinic acetylcholine receptor (α7nAchR) blockade. Specifically, VNS inhibited the pro-inflammatory factors TNF-α, IL-1β, and IL-6 and increased the expression of the anti-inflammatory factors IL-10. Furthermore, we found that VNS promotes the shift of M1-polarized Iba-1^+^/CD86^+^ microglia to M2-polarized Iba-1^+^/CD206^+^ microglia *via* upregulating α7nAchR to alleviate neuroinflammation after SCI.

**Conclusion:**

Our results demonstrated that VNS promotes microglial M2 polarization through upregulating α7nAChR to reduce neuroinflammation, thus improving motor function recovery after SCI. These findings indicate VNS might be a promising neuromodulation strategy for SCI.

## Introduction

Spinal cord injury (SCI) is a common and devastating central nervous system (CNS) disease which often leads to permanent motor function deficits below the injury level. It not only affects the quality of life of individuals, but also brings a heavy burden to the family and society (McDonald and Sadowsky, 2002). But frustratingly, there is still a lack of effective treatment methods for SCI despite a great deal of effort (Fan et al., 2018).

Vagus nerve stimulation (VNS) is a neuromodulation method that has been applied to a variety of CNS diseases, including refractory epilepsy, depression, and migraine (Ben-Menachem et al., 2015). More strikingly, recent studies demonstrated that VNS paired with rehabilitative training can effectively improve the forelimb motor function in rats after SCI (Ganzer et al., 2018; Darrow et al., 2020), but the underlying pathophysiological mechanisms are largely obscure. Over the past two decades, an emerging area of research has been the modulation of immune homeostasis by the nervous system (Zhang et al., 2017). The nervous system was proved to play key roles in controlling immunity and fine-tuning responses to inflammation (Pavlov and Tracey, 2017). Expectedly, VNS has been demonstrated to alleviate inflammation through the cholinergic anti-inflammatory pathway (CAP) in a range of diseases, such as systemic lupus erythematosus and rheumatoid arthritis (Koopman et al., 2016; Aranow et al., 2021). This pathway releases acetylcholine (Ach), which then acts on macrophages through the alpha 7 nicotinic acetylcholine receptor (α7nAchR) to reduce inflammation (Borovikova et al., 2000; Maurer and Williams, 2017). These suggest that VNS might ameliorate SCI by regulating inflammation.

The pathophysiology of SCI, mainly involves the primary and secondary damage mechanisms of tissue damage, while the latter is more crucial for treatment (Tsintou et al., 2015; Ahuja et al., 2017). The mechanisms of secondary injury include inflammation, scar formation, and axonal degeneration (Garcia et al., 2016). As the innate immune cells of the CNS, microglia play a key regulatory role in the secondary inflammatory process after SCI (Graeber et al., 2011; Liu Z. et al., 2020). Activated microglia are divided into pro-inflammatory M1 phenotype and anti-inflammatory M2 phenotype, with M1 aggravating inflammation by secreting inflammatory factors, while M2 secreting anti-inflammatory factors alleviating inflammation (Kigerl et al., 2009; Orihuela et al., 2016). Previous studies have proved that regulating microglia transformation from M1 to M2 can significantly attenuate neuroinflammation and promote functional recovery after SCI (Gao et al., 2019; Liu W. et al., 2020). More importantly, there is evidence that activation of α7nAchR can promote the conversion of M1 microglia to M2 (Zhang et al., 2017). These findings further suggest that VNS might promote microglial polarization to regulate inflammation after SCI.

Therefore, using a rat model of SCI, we have further confirmed the efficacy of VNS in SCI and tested the hypothesis that VNS promotes microglial M2 polarization to reduce neuroinflammation *via* upregulating α7nAChR, thereby improving motor function recovery after SCI.

## Materials and Methods

### Animals

One hundred and ninety-nine female Sprague–Dawley (SD) rats (Army Medical University) weighing 250–300 g were used in this experimentation. All rats were maintained under a 12 h light/dark cycle pathogen-free condition with free access to food and water. Animal use protocols were approved by the Animal Care and Use Committee of the Army Medical University.

### Surgery (Spinal Cord Injury Model and Vagus Nerve Stimulation Cuff Implantation)

The spinal cord injury model was induced by compression injury (Weaver et al., 2001), and then the stimulation electrode was placed under the left cervical vagus nerve. In short, the animals were anesthetized by intraperitoneal injection of pentobarbital (40 mg/kg). Aseptic technique was used to expose the spinal column of rats through a midline incision in the back. After T10 laminectomy, the spinal cord was compressed with a clamp (50 g closing force) for 60 s to induce spinal cord injury. Then place the electrode on the left cervical vagus nerve and draw the wire from the back of the neck in Sham-VNS, VNS, VNS-MLA groups. The stimulation parameters were as follows: 1.0 mA, 0.5 ms, 30 Hz, and 10 min per day for 14 days (Dong and Feng, 2018). All rats were awake during stimulation without strict restraint devices, and the rats could move freely in a box of approximately 25 cm × 15 cm. Rats in the Sham group were subjected to the same operation but without compression and electrode implantation. All rats were given artificial urination twice a day until bladder control was restored.

### Drug

A selective α7nAchR antagonist, methyllycaconitine citrate (MLA, MedChemExpress, United States); or a selective α7nAchR agonist, PNU-282987 (MedChemExpress, United States), was used in this study. MLA and PNU-282987 were dissolved in DMSO and prepared at a dosage of 10 μg in a volume of 20 μl (Zhang et al., 2015). After isoflurane anesthesia (2%), administration of the drugs was performed in rats at the level of L5-L6, which is far from the injury site (T10), using direct transcutaneous intrathecal injection with no impairment on the rat’s normal motor function (Mestre et al., 1994). Thus, the possibility of puncture injury to the spinal cord could be excluded. All injected rats returned to full recovery of activity within 10 min. We injected the rats 30 min before VNS treatment once a day for 14 consecutive days (Li et al., 2020).

### Hindlimb Function Score

Basso-Beattie-Bresnahan (BBB) scores were used to evaluate hindlimb motor function (Basso et al., 1995). The rats were placed on a circular platform with a diameter of 2 m. Each group was scored 1 day before surgery and 1, 3, 7, 10, 14, 21, and 28 days post-injury. The BBB scores with a total score of 21 points were divided into three stages. The range of the first stage for measuring joint activity was 0–7 points, the range of the second stage for measuring gait and the coordination was 8–13 points, and the range of the third stage for measuring fine movements of the claws was 14–21 points. The evaluation was conducted by two independent inspectors who were blind to the treatment plan.

### Electrophysiological Assessment

Motor evoked potentials (MEPs) were used to evaluate the functional integrity of the spinal cord pathway (Chen et al., 2020). Rats were intraperitoneally injected with 1% Pentobarbital Sodium (20 mg/kg). A monopole needle electrode was inserted subcutaneously into the nasal floor as an anode. The other electrode is inserted subcutaneously at the midpoint of the line between the two ears, and its tip contacts the bone as the cathode. The recording electrode was inserted into the gastrocnemius muscle, and the grounding electrode was inserted into the tail subcutaneously. A single electrical pulse (10 mA, 0.1 ms, 1 Hz) stimulated the brain and recorded the amplitude of MEPs.

### Immunofluorescence Staining

At the indicated time points, the animals were deeply anesthetized with sodium pentobarbital (80 mg/kg) and transcardially perfused with sterile 0.9% normal saline followed by treatment with cold 4% paraformaldehyde. Immediately after perfusion, the spinal cord tissue at the lesion site was dissected, and placed in paraformaldehyde overnight. Subsequently, the tissue is dehydrated in paraformaldehyde solutions with graded concentrations of sucrose (10, 20, and 30%), embedded in optimum cutting temperature (OCT) compound, cut into 18 μm frozen sections with a microtome (Leica, Wetzlar, Hesse, Germany).

The cryofixed sections of the spinal cord were thawed at room temperature for 30 min. The sections were washed three times (for 5 min each) with PBS. Then permeabilized with 0.5% Triton X-100 in PBS for 15 min at room temperature, and blocked with 10% goat serum for another 2 h. incubated overnight at 4°C with the following primary antibodies. and incubated overnight with a primary antibody at 4°C. After washing with PBS the next day (3 times for 10 min each), the sections were incubated with the appropriate secondary antibody for 1.5 h at room temperature and then washed with PBS. The nuclei were stained with 4’,6-diamidino-2-phenylindole (DAPI; Boster, Wuhan, China) for 5 min, and the sections were washed with PBS and sealed with an antifluorescence quencher (Boster, Wuhan, China). Images were captured under a Zeiss lsm780 confocal microscope.

The primary antibodies used in the experiment are as follows: mouse anti-ionized calcium-binding adaptor molecule (Iba-1; 1:200; GeneTex, United States), rabbit anti-Iba-1 (1:200; GeneTex, United States), mouse anti-glial fibrillary acid protein (GFAP; Boster, Wuhan, China), rabbit anti-Laminin (1:300; Sigma-Aldrich, MO, United States), mouse anti-CD86 (1:200; Santa Cruz, CA, United States), mouse anti-CD206 (1:150; Santa Cruz, CA, United States), rat anti-α7AChR (1:200; Santa Cruz, CA, United States).

### Hematoxylin-Eosin Staining

The Tissues were obtained and fixed in 4% paraformaldehyde as described above. The tissue was dehydrated in xylene and gradient alcohol solutions, embedded in paraffin, and cut into 5μm sections with a slicer. A series of sections were stained with hematoxylin for 1 min, double-rinsed in distilled water, differentiated in 1% hydrochloric acid, double-rinsed in distilled water, and stained with eosin for 2 min. Then measure the cavity area of spinal cord tissue in each group.

### Nissl Staining

Nissl staining was performed to assess neuronal cell damage by measuring dark neurons (Kupats et al., 2020). The frozen sections of 28 days post-injury were incubated with 1% cresyl violet (Beyotime, Shanghai, China) according to the manufacturer’s protocol. In brief, the frozen sections were washed twice with distilled water and incubated with 1% cresyl violet for 10 min. After washing twice with distilled water, the sections were dehydrated in 95% alcohol, cleared in xylene, and sealed with neutral balsam.

### Enzyme-Linked Immunosorbent Assay

Since peripheral blood serum cannot accurately reflect the inflammatory status of the spinal cord, tissues were obtained 3 days after SCI to evaluate the expression levels of pro-inflammatory cytokines including TNF-α, IL-1β, and IL-6 and anti-inflammatory cytokines IL-10 in the injured spinal cord (Wang et al., 2019; Liu W. et al., 2020). Spinal cord tissue was homogenized in radioimmunoprecipitation assay (RIPA) lysis buffer, which included 1 mM EDTA, 1 mM PMSF, 150 mM NaCl, 10 mM Tris (pH 8.0), and protease inhibitor and incubated with shaking for 1 h at 4°C. Collected the supernatant after centrifugation (12,000 rpm, for 10 min at 4°C). and stored at -80°C. Measured the cytokine concentration using Enzyme-Linked Immunosorbent Assay (ELISA) kits according to the manufacturer’s protocol.

### Quantitative Reverse Transcription-Polymerase Chain Reaction

Total RNA was extracted from spinal cord tissues with TRIzol (Takara, Dalian, China) following the manufacturer’s protocol. The purity and concentration of total RNA in each sample were then assessed using an ultraviolet-visible light spectrophotometer (NanoDrop 2000, Thermo Fisher Scientific, Waltham, MA, United States). Complementary DNA (cDNA) was prepared from a total of RNA 1μg by using reverse transcription kits (AGbio, ChangSha, China), and then quantitative reverse transcription-polymerase chain reaction (qRT-PCR) was carried out using SYBR Green Premix Pro (AGbio, ChangSha, China). CFX-96 Real-Time PCR Detection System (Bio-Rad, Hercules, CA, United States) was used to perform the following reaction: initial denaturation at 95°C for 30 s, 40 cycles of 95°C for 5 s, and 60°C for 30 s, following a melt curve. The expression level of housekeeping gene GAPDH was used as an internal control, and the relative expression levels were evaluated using the 2^–ΔΔCT^ method. The primer sequences are listed in Table 1.

**TABLE 1:** Forward and reverse primer sequences for quantitative reverse transcription-polymerase chain reaction (qRT-PCR).

Rat gene	Forward primer (5′-3′)	Reverse primer (5′-3′)
Cd11b	GTCAGCGTGGTCTTCTGGAT	GACACTTGAGAGGTTCTGGGA
iNOS	CCCTTCAATGGTTGGTACATGG	ACATTGATCTCCGTGACAGCC
CD68	ATCTCTCTTGCTGCCTCTCATC	GGCTGGTAGGTTGATTGTCGT
CCL-22	GGGCAGGAAGGACCATACAAA	CCAGGGAAGCAAGAGTGAGTT
TGF-β	CTGCTGACCCCCACTGATA	AAGCCCTGTATTCCGTCTCC
GAPDH	ATGGTGAAGGTCGGTGTGA	CTCCACTTTGCCACTGCAA

### Western Blotting

Total protein was extracted using RIPA lysis buffer (including protease inhibitor cocktail). After the protein concentration of each sample was determined by a bicinchoninic acid (BCA) kit, the loading buffer (5×; Boster, Wuhan, China). was added and boiled for 5 min for Western blotting. Equal amounts of protein from each sample were separated using 12.5% SDS-PAGE and transferred to polyvinylidene fluoride (PVDF) membranes. The membranes were blocked with 5% skim milk at room temperature for 1.5 h and then incubated with a monoclonal rat anti-α7nAChR antibody (1:200; Santa Cruz, CA, United States), and a monoclonal mouse anti-GAPDH antibody (1:10,000; ZenBioScience, China) overnight at 4°C. After incubation with the appropriate HRP-conjugated secondary antibodies at room temperature for 1 h, the membranes were scanned using a Fusion Edge system (Vilber, FR), and the density of the results was quantified using ImageJ software (NIH, Bethesda, MD, United States). The GAPDH were used as internal controls.

### Statistical Analyses

All Statistical data are presented as means values with standard error of the mean and analyzed by GraphPad Prism 8 (GraphPad, La Jolla, CA, United States). The statistic of BBB scores was performed by two-way analysis of variance (ANOVA) with repeated measures followed by Tukey’s multiple comparison test. Other statistical data were analyzed by one-way ANOVA followed by Tukey’s multiple comparisons test and Student’s *t*-test. A value of <0.05 was considered to be statistically significant.

## Results

### Vagus Nerve Stimulation Improves Long-Term Outcomes After Spinal Cord Injury

**FIGURE 1:**
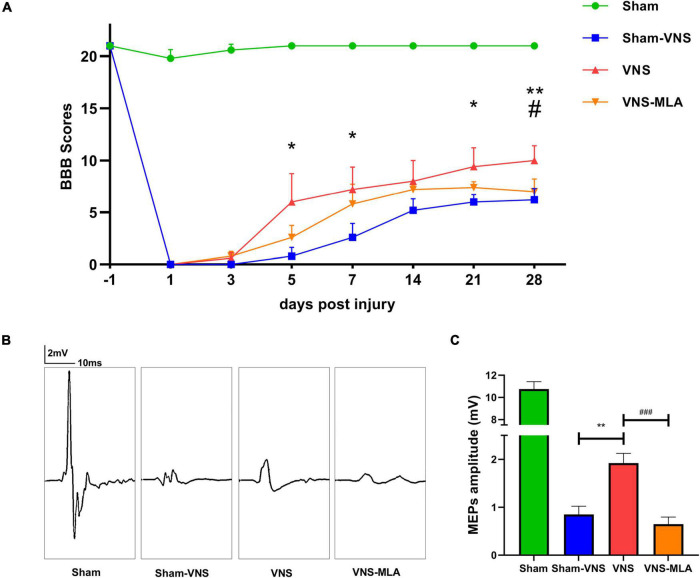
Vagus nerve stimulation (VNS) promoted functional behavioral recovery after spinal cord injury (SCI). **(A)** Basso-Beattie-Bresnahan (BBB) was used to functionally grade the rats in the Sham, Sham-VNS, VNS, and VNS-MLA groups up to 28 days post-injury, *n* = 5 per group. **(B,C)** Representative recordings and analysis of motor evoked potentials (MEPs) in different groups at day 28 post-injury, *n* = 5 per group, scale: 2 mV/10 ms. *P < 0.05, **P < 0.01 between the Sham and Sham-VNS groups, ^#^*P* < 0.05, ^##^*P* < 0.01 between the VNS and VNS-MLA groups.

We assessed the therapeutic effect of VNS after SCI. We used BBB motor function scores and MEPs analysis to assess motor function for 28 days after SCI. Normal motor function was scored as 21 points. As shown in Figure 1A, the BBB scores were significantly higher in the VNS group than in the Sham-VNS and VNS-MLA groups at 28 days after injury, and the final scores of Sham, Sham-VNS, VNS, and VNS-MLA groups were 21.0 ± 0.0, 6.2 ± 1.1, 10.0 ± 1.4 and 7.0 ± 1.2, respectively. To further study motor functional behavioral recovery, electrophysiological analyses were applied. As shown in Figures 1B,C, MEPs amplitudes were higher in the VNS group than in the Sham-VNS and VNS-MLA group at 28 days post-injury, indicating that hindlimbs exhibited better recovery of electrophysiological functions with the treatment of VNS. Taken together, these results indicate that VNS could promote functional behavioral recovery following SCI in rats and that these beneficial effects can be attenuated by MLA.

### Vagus Nerve Stimulation Provides a Beneficial Microenvironment for Neurons

**FIGURE 2:**
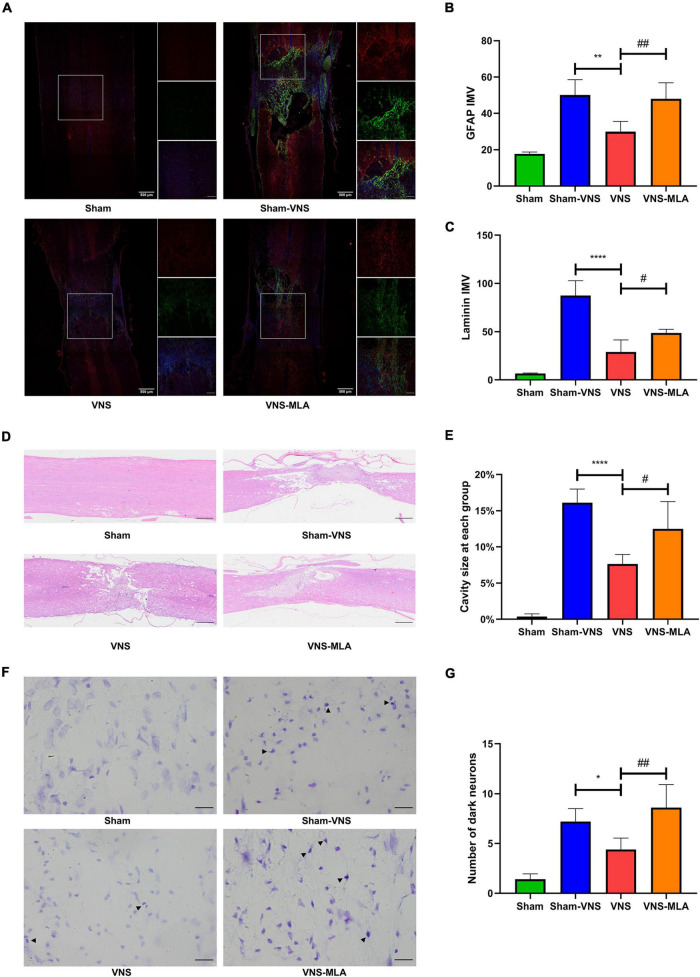
Vagus nerve stimulation (VNS) decreases spinal cord tissue damage. **(A–C)** Representative Immunofluorescence (IF) staining images of each group post injure 28 days, and bar charts show the fluorescence intensity mean value (IMV) for GFAP and Laminin, *n* = 5 per group, scale bar = 500 or 200 μm. **(D,E)** Representative Hematoxylin-eosin (HE) staining images 28 days after injury and quantification data of cavity necrotic tissue in each group, *n* = 5 per group, scale bar = 500 μm. **(F,G)** Representative Nissl staining images 28 days after injury and quantification of the numbers of Nissl-stained dark neurons in each group, *n* = 5 per group, scale bar = 20 μm. *P < 0.05, **P < 0.01, and ****P < 0.0001 between the Sham and Sham-VNS groups, ^#^*P* < 0.05, ^##^*P* < 0.01 between the VNS and VNS-MLA groups.

To assess the effect of VNS on scar formation, we assessed scar formation (glial and fibrotic) 28 days after SCI. The lesions of Sham-VNS and VNS-MLA contain large amounts of laminin, which is a sign of fibrotic scars that are thought to hinder axon regeneration, but the content in lesions of VNS rats is much lower (Figures 2A,B). And The expression of GFAP indicating glial formation was also lower than that of Sham-VNS and VNS-MLA rats, indicating that VNS reduced glial scar formation (Figures 2A,C). Furthermore, VNS treatment significantly decreased the damage caused by SCI, alleviating the cavity of necrotic tissue (Figures 2D,E). Nissl-stained dark neurons were also conducted to confirm neuronal damage, and the number of dark neurons in the VNS group was significantly reduced compared to the Sham-VNS group and the VNS-MLA group (Figures 2F,G). Taken together, VNS could exert a neuroprotective effect on SCI in rats.

### Vagus Nerve Stimulation Reduces the Activation of Microglia in the Spinal Cord Injury Site

**FIGURE 3:**
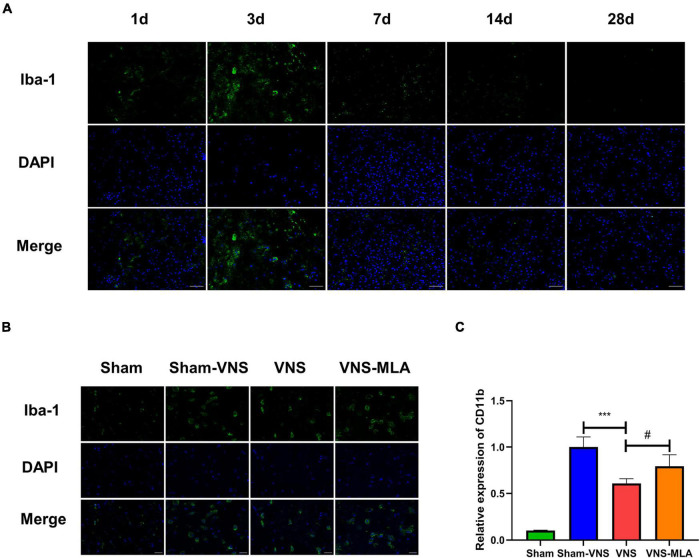
Vagus nerve stimulation (VNS) reduced microglial activation. **(A)** Immunofluorescence staining showed the amounts of microglia in spinal cord tissues at different time points, scale bar = 50 μm. **(B)** Iba-1 staining in microglia in the spinal cord sections on day 3 after spinal cord injury (SCI). Activated microglia exhibited significant cellular hypertrophy and retraction of processes after SCI, while resting microglia displayed smaller somata and processes, scale bar = 20 μm. **(C)** Quantitative reverse transcription-polymerase chain reaction (qRT-PCR) demonstrated the gene levels of CD11b in the spinal cord of different groups on day 3 after SCI. *n* = 4 per group, ***P < 0.001 VNS vs. Sham-VNS group. ^#^*P* < 0.05 VNS vs. VNS-MLA group.

As mentioned above, microglial activation is a marker of spinal cord injury. We use an anti-Iba-1 antibody for immunofluorescence, and the results showed that microglia exhibited marked cellular hypertrophy and retraction of cytoplasmic processes after SCI (Figure 3B), especially on the 3rd day (Figure 3A). As a molecular marker of microglia, CD11b has been widely used for microglial activation. The gene level of CD11b was decreased in the VNS group, while MLA promoted CD11b gene expression (Figure 3C). Microglial activation is at peak levels at 3 days after SCI. Thus, the time point 3 days after injury was selected for subsequent investigations.

### Vagus Nerve Stimulation Modulates Neuroinflammation at the Injury Site

**FIGURE 4:**
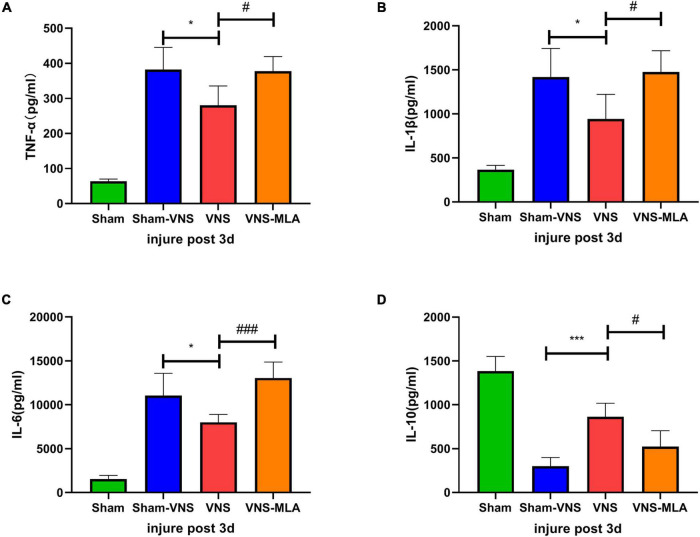
Vagus nerve stimulation (VNS) promotes changes in neuroinflammation after spinal cord injury (SCI). **(A–C)** Changes in the levels of the pro-inflammatory cytokines TNF-α, IL-1β, and IL-6, *n* = 5 per group. **(D)** Changes in the levels of the anti-inflammatory cytokines IL-10, *n* = 5 per group. *P < 0.05, ***P < 0.001 VNS vs. Sham-VNS group. ^#^*P* < 0.05, ^###^*P* < 0.001 VNS vs. VNS-MLA group.

Three days after injury, we measured the concentration of pro-inflammatory cytokines TNF-α, IL-1β, and IL-6 and anti-inflammatory cytokine IL-10 in the spinal cord tissues in the different groups by ELISA. The results showed that administration of VNS could significantly decrease the concentrations of the pro-inflammatory cytokines and elevate the concentrations of the anti-inflammatory cytokines compared with the Sham-VNS group (Figures 4A–D). However, VNS combined with MLA could promote the secretion of pro-inflammatory cytokines and inhibit the secretion of anti-inflammatory cytokines when compared with the VNS group (Figures 4A–D).

In addition, we performed the same experiment 14 days after SCI. Consistent with the 3-day results, VNS significantly decreased the concentration of pro-inflammatory cytokines and increased the concentration of anti-inflammatory cytokines compared with the Sham-VNS group. This beneficial effect was similarly hindered by the administration of MLA (Supplementary Figures 1A–D).

### Vagus Nerve Stimulation Promotes Microglial M2 Polarization

**FIGURE 5:**
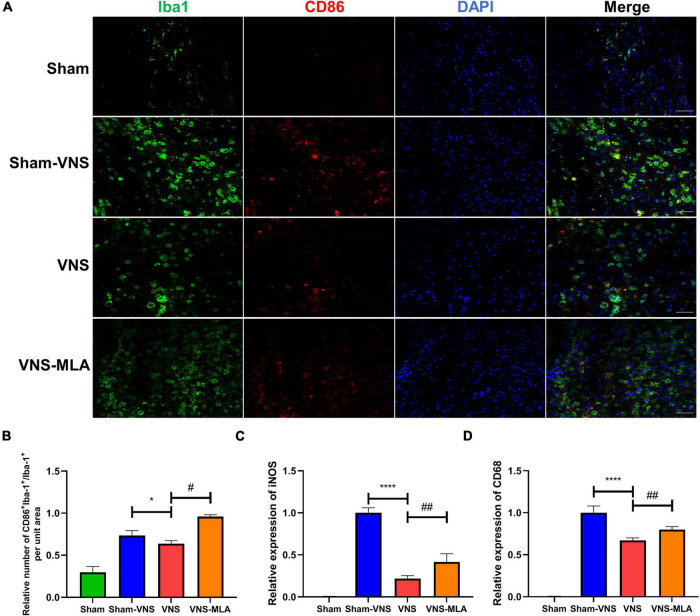
Vagus nerve stimulation (VNS) significantly decreases CD86 production in microglia. **(A)** Representative images showing Iba-1+/CD86+IF staining after spinal cord injury (SCI), *n* = 5 per group, scale bar = 50 μm. **(B)** Quantitative analysis of the results in panel a, *n* = 5 per group. **(C,D)** Relative mRNA expression levels of iNOS and CD68 in all groups, as detected by qRT-PCR, *n* = 4 per group. *P < 0.05, ****P < 0.0001 VNS vs. Sham-VNS group. ^#^*P* < 0.05, ^##^*P* < 0.01 VNS vs. VNS-MLA group.

**FIGURE 6:**
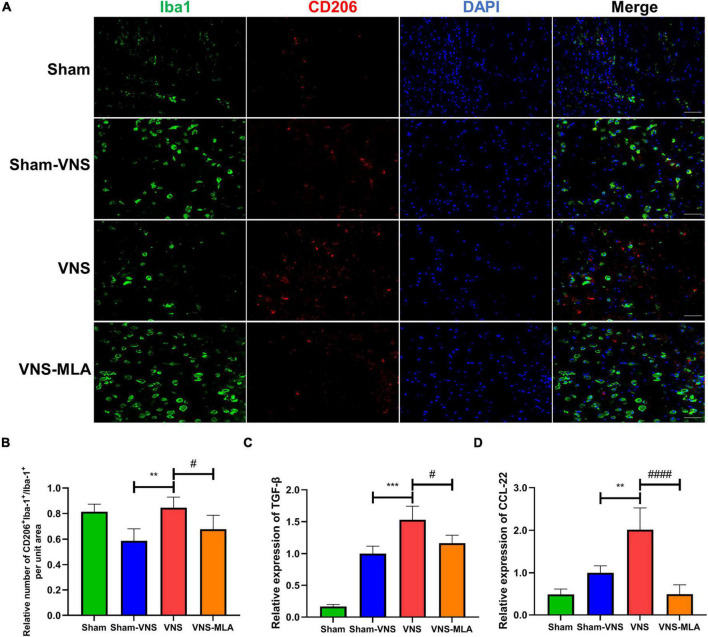
Vagus nerve stimulation (VNS) significantly increases CD206 expression in microglia. **(A)** Representative images showing Iba-1+/CD206+IF staining after spinal cord injury (SCI), *n* = 5 per group, scale bar = 50 μm. **(B)** Quantitative analysis of the results in panel a, *n* = 5 per group. **(C,D)** Relative mRNA expression levels of Arg-1 and IL-4 in all groups, as detected by qRT-PCR, *n* = 4 per group). **P < 0.01, ***P < 0.001 VNS vs. Sham-VNS group. ^#^*P* < 0.05, ^####^*P* < 0.0001 VNS vs. VNS-MLA group.

As microglia can have two different phenotypes, we, therefore, queried whether VNS could polarize microglia from the M1 phenotype toward the M2 after SCI. The gene expression of M1 (iNOS, CD68) and M2 (CCL-22, TGF-β) was analyzed by qRT-PCR. As shown in Figures 5C,D, 6C,D, the M2 gene expression in the VNS groups was significantly increased, and M1 gene expression decreased compared with the Sham-VNS group. Meanwhile, M1 gene expression was higher and M2 gene expression lower in the VNS-MLA group compared with the VNS group. Furthermore, we evaluated the characteristic polarization of microglia after SCI in different groups using the representative M1-associated CD86 and M2-associated CD206 markers for double immunofluorescent staining together with Iba-1, which detects microglia in the injured spinal cord. The results showed that the ratio of CD86^+^/Iba-1^+^ (M1) was decreased, while the ratio of CD206^+^/Iba-1^+^ (M2) was increased in the VNS group compared with the Sham-VNS group, which was suppressed by using MLA (Figures 5A,B, 6A,B). We also evaluated the CD86 and CD206 markers at 14 days, and the results were consistent with that at 3 days (Supplementary Figures 2A,B, 3A,B).

### Vagus Nerve Stimulation Improves Recovery of Function and Tissue After Spinal Cord Injury Requires α7nAChR

**FIGURE 7:**
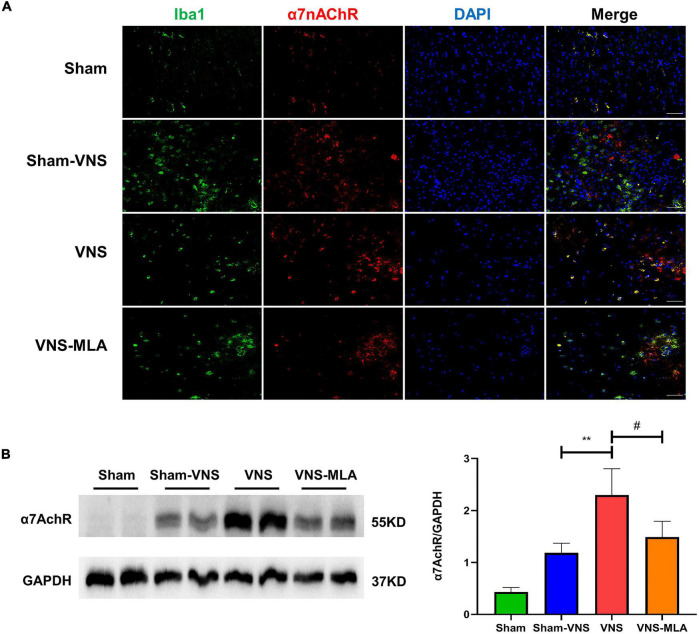
Vagus nerve stimulation (VNS) improves recovery *via*α7Acher. **(A)** α7nAChR labeled cells (green) express Iba-1 (red) in the spinal cord, *n* = 5 per group, scale bar = 50 μm. **(B)** Western blot analysis of α7nAChR expression and the spinal cords were collected at 3 days after injury, *n* = 4 per group. **P < 0.01 VNS vs. Sham-VNS group. ^#^*P* < 0.05 VNS vs. VNS-MLA group.

To confirm that VNS treats spinal cord injury *via*α7nAChR, we tested the expression of α7nAChR in the spinal cord at 3 days. The cellular localization of α7nAChR was investigated using immunostaining analysis. In injured spinal cords, α7nAChR was colocalized with Iba-1 in microglia (Figure 7A). As shown in Figure 7B, VNS increase the α7nAChR expression in SCI rats, and MLA prevented the increase in α7nAChR expression mediated by VNS. Similar to 3 days, VNS increase the α7nAChR expression in SCI rats after 14 days, and MLA prevented it (Supplementary Figures 4A,B). With reference to MLA hindering the function and tissue recovery of VNS, we can conclude that the treatment of VNS on SCI requires α7nAChR.

As we observed that VNS promotes microglial M2 polarization requires α7nAChR. To further explore the mechanism, we injected the α7nAChR agonist PNU-282987 and vehicle into SCI rats in the same way as MLA, and the results showed that activation of α7nAChR promotes the M2 polarization of microglia at 3 days (Supplementary Figures 5A–D), and 14 days (Supplementary Figures 5E–H).

## Discussion

Current treatments for SCI focus on secondary damage and suppression of neuroinflammation, to create a favorable immune microenvironment for neuron survival and axon regeneration (Young, 1993; Jain et al., 2015). Although great efforts have been made, the current effects of SCI treatment are still limited. In our study, we saw the promotion of VNS in functional and tissue recovery in rats through 4 weeks of observation. We demonstrated that VNS reduced scar formation and improved behavioral scores after SCI. We then explored the underlying mechanism, and found that VNS promotes microglial M2 polarization to alleviate neuroinflammation through upregulating α7nAChR after SCI.

As the major immune cells of CNS, microglia are rapidly activated in response to SCI (Gensel et al., 2011). The excessive activation of microglia will aggravate the inflammatory response and aggravate secondary damage (David and Kroner, 2011; Swaroop et al., 2018). And studies have also illustrated that microglia contribute to the repair of injury in the CNS, including SCI (Block et al., 2007; David and Kroner, 2011). Microglia can assume the following two phenotypes: a classically activated pro-inflammatory (M1) phenotype that secretes pro-inflammatory cytokines including TNF-α, IL-6, and IL-1β, which are harmful to neurogenesis, and an alternatively activated anti-inflammatory (M2) phenotype that secretes anti-inflammatory cytokines including TGF-β and IL-10, which are favorable to neurogenesis (Kigerl et al., 2009; Crain et al., 2013; Orihuela et al., 2016). Compared with the immune response in peripheral tissue injury, the polarization of microglia post-SCI is dominated by M1 and lasts longer (Kigerl et al., 2009; Kroner et al., 2014; Wang et al., 2015). Thus the proper transition of M1 microglia to M2 would reduce neuroinflammation after SCI (Fan et al., 2019; Liu W. et al., 2020).

There is evidence that the α7nAChR was identified as a major component of the immune modulatory role of the CAP in macrophages (Wang et al., 2004, 2003). The acetylcholine receptors in spinal cord cells were selectively blocked by the intramedullary injection of MLA, but the macrophages in the reticuloendothelial system were not affected. Compared with VNS, VNS-MLA showed worse motor function recovery and more tissue damage. Thus, it can be proven that α7nAChR in the spinal cord is the key target of VNS in the anti-neuroinflammation effect after SCI. Therefore, it can be concluded that upregulating the expression of α7nAChR reduces neuroinflammation and promotes functional recovery after SCI. These effects on microglia are produced by α7nAChR in the spinal cord, not only from the systemic anti-inflammatory effects of VNS leading to a reduction in the overall neuroinflammatory burden.

Compared with the Sham-VNS group, VNS increased the number of M2 microglia. Zhang et al. (2017) showed that Ach promoted the transformation of M1 microglia to the M2 phenotype through the JAK2/STAT3 pathway by activation of α7nAChR. A previous study showed that administration of IL-4 significantly reduced tissue damage and promoted functional recovery by shifting microglia to the M2 phenotype after SCI (Francos-Quijorna et al., 2016). Further, direct transplantation of IL-4-induced M2 microglia promotes functional recovery in mice after SCI (Kobashi et al., 2020). Another study confirmed that M2 microglia can enhance neurite length in primary neurons by secreting neurotrophic factors (Tanaka et al., 2015). Considering that the M2 phenotype has anti-inflammatory and neuroprotective effects, the treatment of differentiated microglia from M1 to M2 phenotype can be considered in traumatic neurological diseases (David and Kroner, 2011; Li et al., 2017; Sun et al., 2018). Also, the immune response generated by microglia has been shown to contribute to the formation and expansion of the cavity after SCI (Fitch et al., 1999), and the destructive effects of activated microglia/macrophages are mainly attributed to their M1 subgroup (Fan et al., 2016). In this study, VNS was shown to promote microglial M2 polarization and produce anti-inflammatory cytokines in rats. Based on these results, we can conclude that VNS could be a promising effective treatment to improve functional behavioral recovery by shifting the microglial M1/2 phenotype following SCI in rats. In this experiment, the average BBB scores of the Sham-VNS group was about 6 points at 28 days, which mainly showed as extensive movement of two joints and slight movement of the third, while the VNS group was 10 points, mainly showed as occasional weight supported plantar steps without forelimb-hindlimb coordination. And at this time, rats can complete the action of active foraging, which is of great significance for the survival of SCI rats. The increase of the above scores is the performance that VNS promoted the improvement of hindlimb motor function in SCI rats. The deficiency in this experiment is that there were not a sufficient number of animal samples for BBB scores, although the combination of molecular and histological methods improved the reliability of BBB scores.

Vagus nerve stimulation triggers the release of pro-plasticity factors, including norepinephrine, acetylcholine, serotonin, brain-derived neurotrophic factor, and fibroblast growth factor (Hays, 2016; Hulsey et al., 2016, 2017). Previous research has shown that closed-loop VNS enhances recovery by strengthening the synaptic connections from the remaining motor networks to the forelimb grasping muscles after SCI (Ganzer et al., 2018). Combined with our research, continuous VNS combined with precisely timed closed-loop stimulation may amplify the recovery effect. Although VNS has its adverse events (AEs), some of which are caused by surgical procedures (Ohemeng and Parham, 2020), non-invasive or minimally invasive treatments have been developed, including transcutaneous auricular VNS (taVNS) and percutaneous VNS (pVNS) (Huffman et al., 2019; Aranow et al., 2021). So the indications of taVNS and pVNS may be expanded in SCI treatment due to the safety, but their therapeutic effect on spinal cord injury still needs to be studied.

In this experiment, we stimulated the rats for 14 consecutive days, which runs through the acute phase of secondary spinal cord injury and is also a key process of early anti-inflammatory. However, the late stage of spinal cord injury is a continuous and chronic process. This experiment is limited to the lack of long-term observation of the anti-neuro-inflammatory effects of VNS, and no knowledge of the benefits of receiving VNS after the acute phase of spinal cord injury. Clinically, in other diseases, it is more common for patients to receive VNS treatment in the later stage.

## Conclusion

Our present study confirmed that VNS reduces scar formation and improves motor function recovery after SCI. Moreover, we further demonstrated that VNS promotes microglial M2 polarization through upregulating α7nAChR to reduce neuroinflammation after SCI. Therefore, our results indicate that VNS might be a promising neuromodulation strategy for SCI treatment.

## Data Availability Statement

The original contributions presented in the study are included in the article/Supplementary Material, further inquiries can be directed to the corresponding author/s.

## Ethics Statement

The animal study was reviewed and approved by the Animal Care and Use Committee of Army Medical University.

## Author Contributions

JMH and HL contributed for the ideas and design of the experiments, and reviewing manuscript. HC, ZF, LM, and WD performed the experiments. ZF, MT, and JH contributed for processing the data. HC, QG, and DZ contributed for making charts and writing the manuscript. All authors contributed to the article and approved the submitted version.

## Conflict of Interest

The authors declare that the research was conducted in the absence of any commercial or financial relationships that could be construed as a potential conflict of interest.

## Publisher’s Note

All claims expressed in this article are solely those of the authors and do not necessarily represent those of their affiliated organizations, or those of the publisher, the editors and the reviewers. Any product that may be evaluated in this article, or claim that may be made by its manufacturer, is not guaranteed or endorsed by the publisher.
