# Supplementary material for: Vagus Nerve Stimulation Reduces Neuroinflammation Through Microglia Polarization Regulation to Improve Functional Recovery After Spinal Cord Injury
Source: Front Neurosci. 2022 Apr 7;16:813472. doi: 10.3389/fnins.2022.813472 (PMC9022634; doi:10.3389/fnins.2022.813472)
Supplement: Supplementary file 1 [file Data_Sheet_1.docx]

Supplementary Figure Legends

Supplementary Fig. 1 VNS promotes changes in neuroinflammation after SCI at day 14. (**A**-**C**) Changes in the levels of the pro-inflammatory cytokines TNF-α, IL-1β, and IL-6, n = 5 per group. (**D**) Changes in the levels of the anti-inflammatory cytokines IL-10, n = 5 per group. *P<0.05, **P<0.01 VNS versus Sham-VNS group. #P< 0.05, ##P< 0.01, ###P<0.001 VNS versus VNS-MLA group.

Supplementary Fig. 2 VNS significantly decreases CD86 production in microglia at day 14. (**A**) Representative images showing Iba-1+/CD86+ IF staining after SCI, n=5 per group, scale bar = 50 μm. (**B**) Quantitative analysis of the results in panel a, n = 5 per group. **P<0.01 VNS versus Sham-VNS group. ##P< 0.01 VNS versus VNS-MLA group.

Supplementary Fig. 3 VNS significantly increases CD206 expression in microglia at day 14. (**A**) Representative images showing Iba-1+/CD206+ IF staining after SCI, n=5 per group, scale bar = 50 μm. (**B**) Quantitative analysis of the results in panel a, n = 5 per group. ****P<0.0001 VNS versus Sham-VNS group. ###P<0.001 VNS versus VNS-MLA group.

Supplementary Fig. 4 VNS promotes α7nAChR expression at day 14. (**A**, **B**) Western blot analysis of α7nAChR expression and the spinal cords were collected at 14d after injury, n = 4 per group. *P<0.05 VNS versus Sham-VNS group. ##P< 0.01 VNS versus VNS-MLA group.

Supplementary Fig. 5 Post-SCI administration of the α7nAChR agonist PNU-282987 promotes microglial M2 polarization at 3 and 14 days. (**A**-**D**) Representative images showing Iba-1+/CD86+ and Iba-1+/CD206+ IF staining at 3 days after SCI, and quantitative analysis of the results in panel a and c, respectively, n = 5 per group, scale bar = 50 μm. (**E**-**H**) Representative images showing Iba-1+/CD86+ and Iba-1+/CD206+ IF staining at 14 days after SCI, and quantitative analysis of the results in panel e and g, respectively, n = 5 per group, scale bar = 50 μm. *P<0.05, **P<0.01 Vehicle versus PNU group. PNU= PNU-282987.
