# Supplementary figures and images for: Vagus Nerve Stimulation Reduces Neuroinflammation Through Microglia Polarization Regulation to Improve Functional Recovery After Spinal Cord Injury
Source: Front Neurosci. 2022 Apr 7;16:813472. doi: 10.3389/fnins.2022.813472 (PMC9022634; doi:10.3389/fnins.2022.813472)

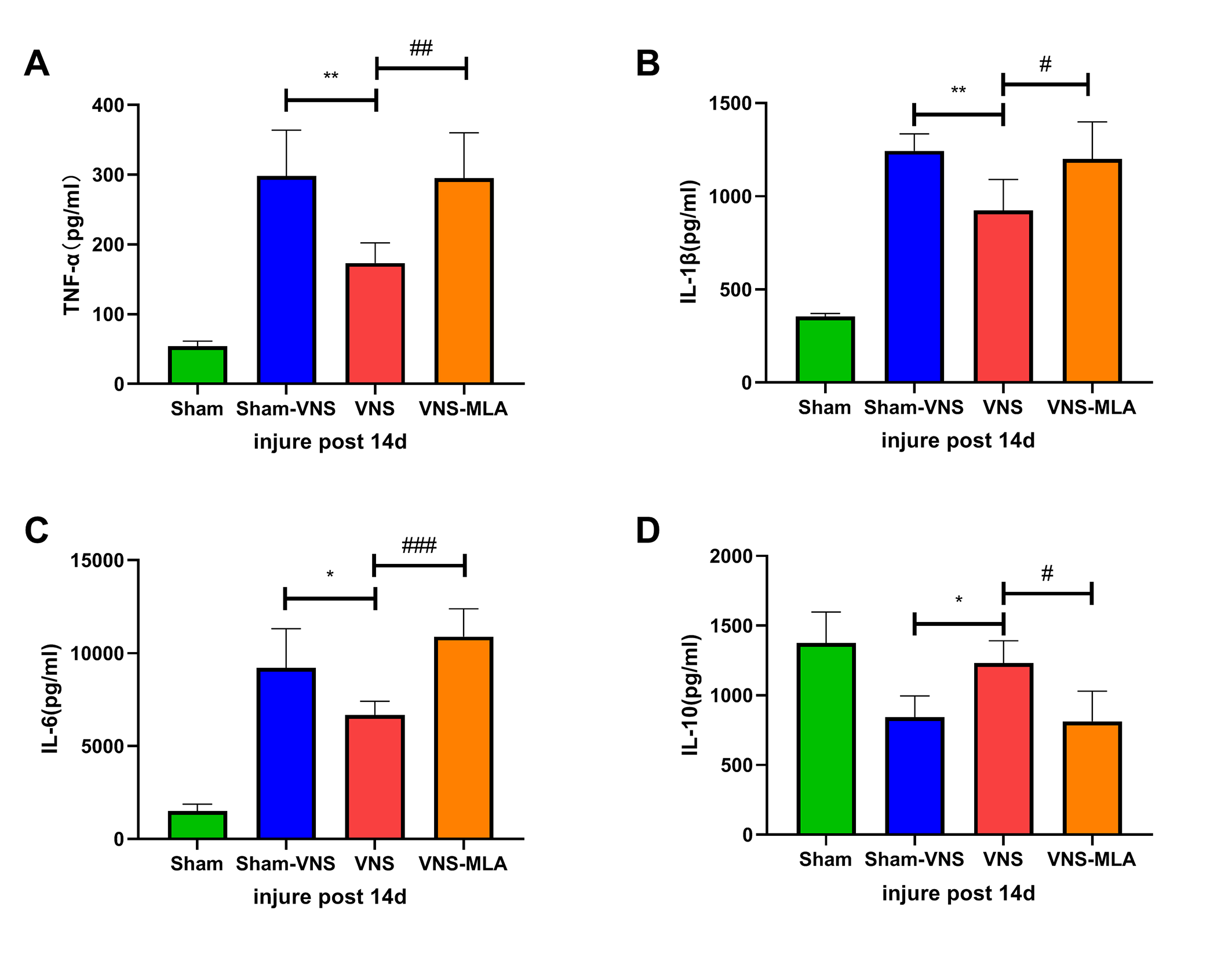

Supplement: Supplementary file 2 [file Image_1.TIF]

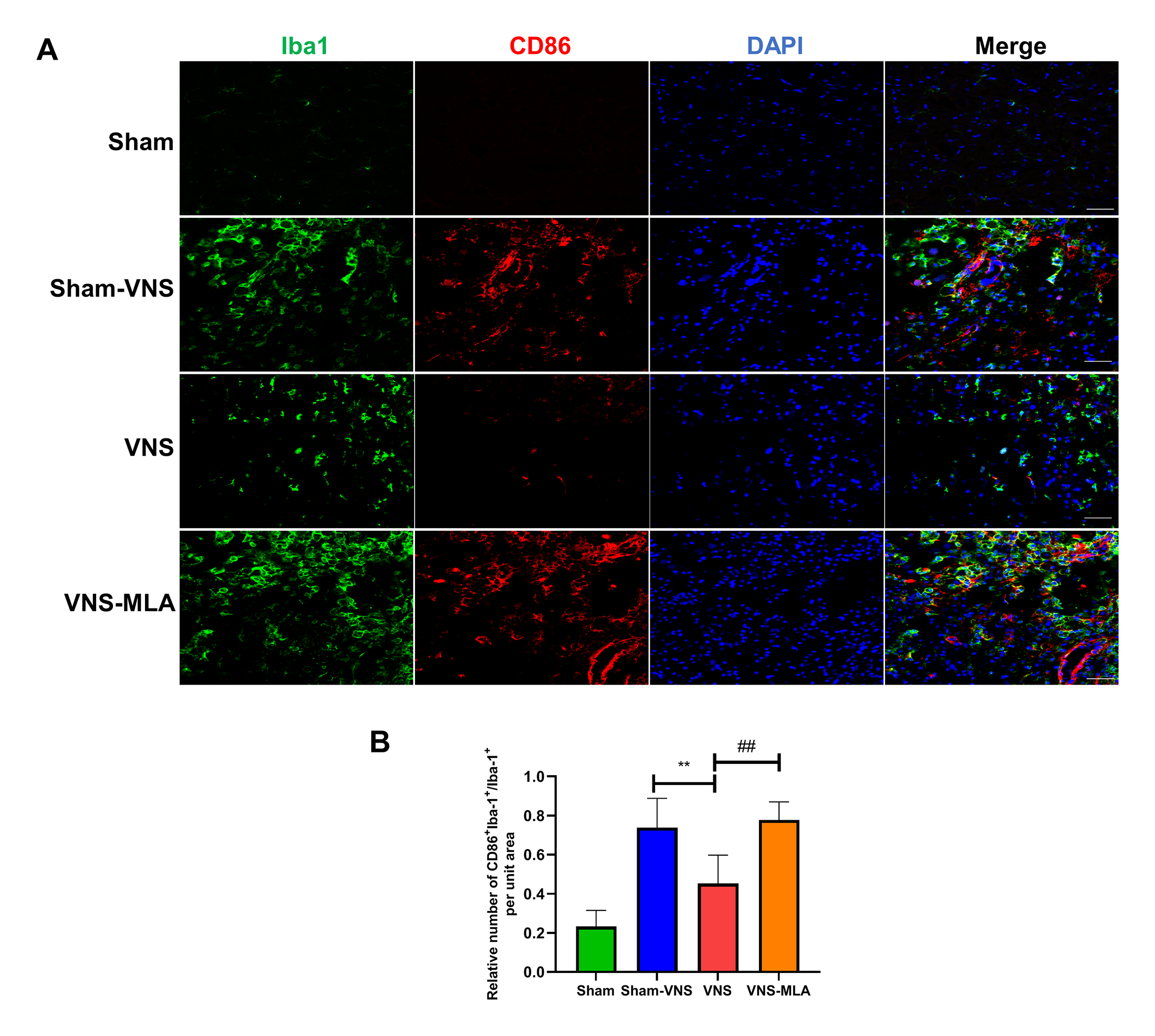

Supplement: Supplementary file 3 [file Image_2.TIF]

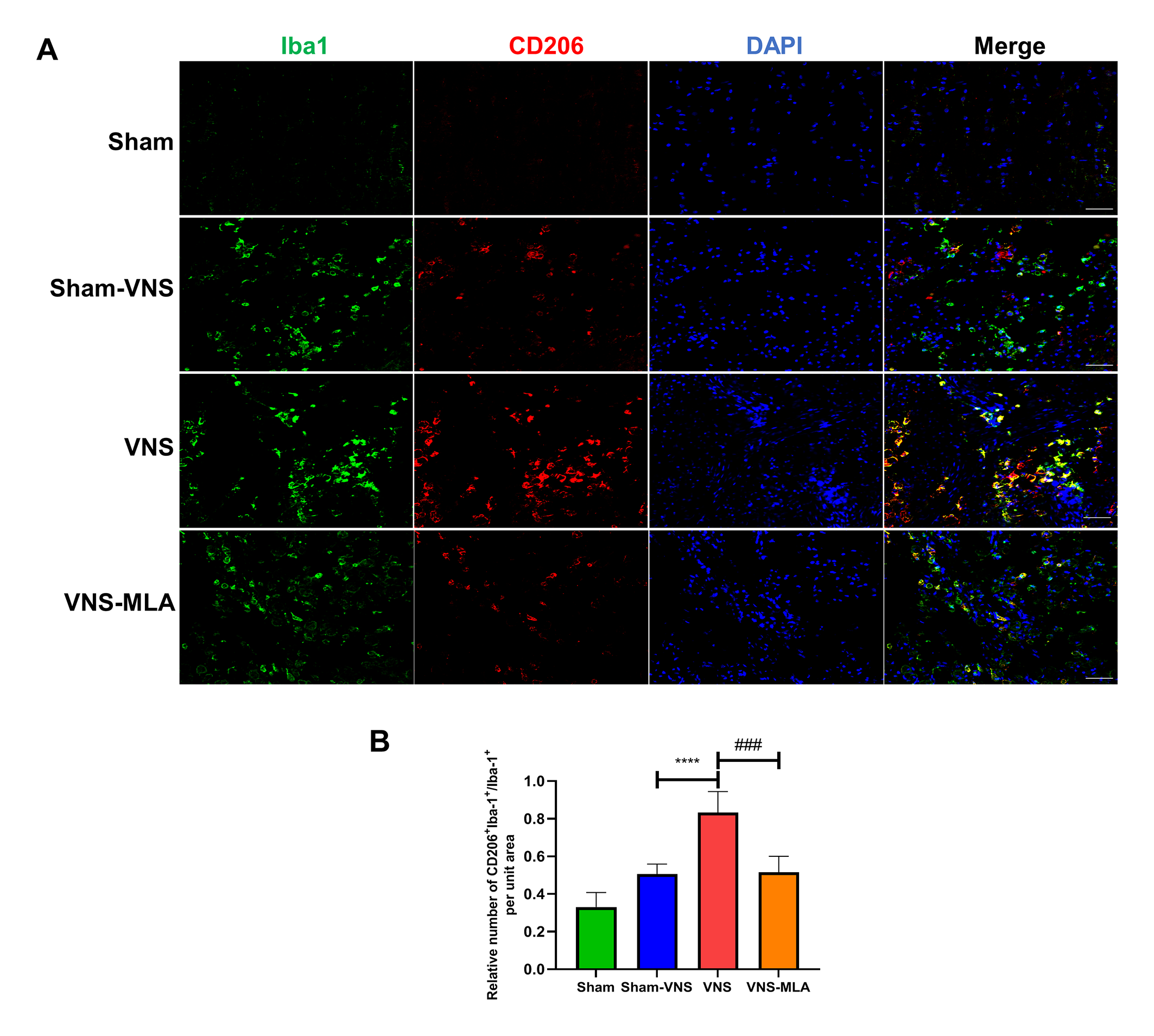

Supplement: Supplementary file 4 [file Image_3.TIF]

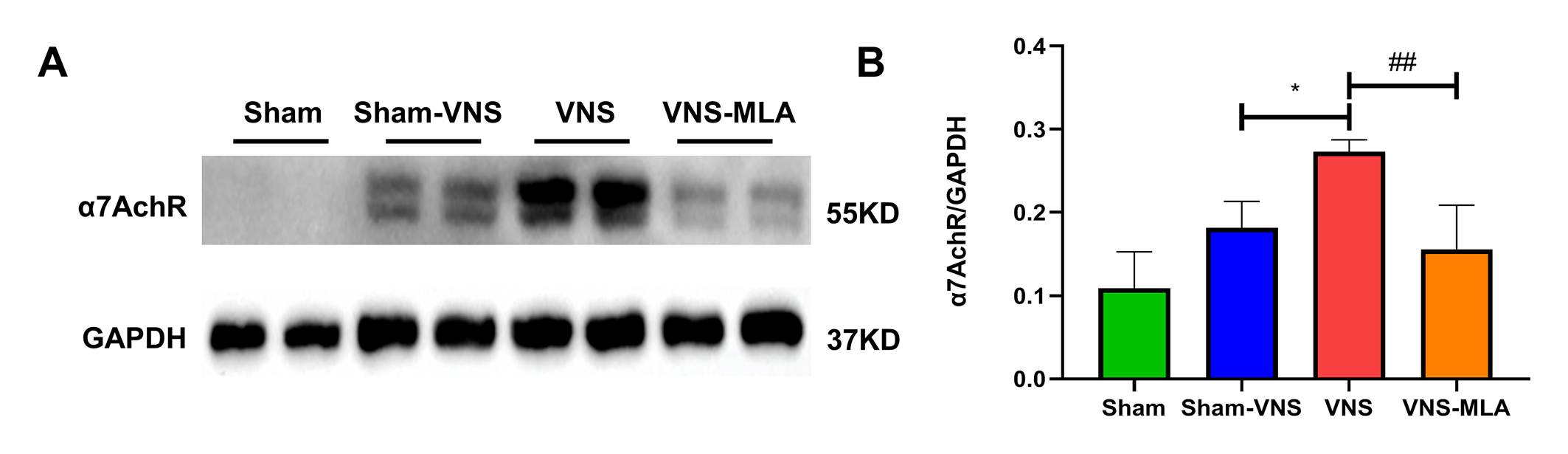

Supplement: Supplementary file 5 [file Image_4.TIF]

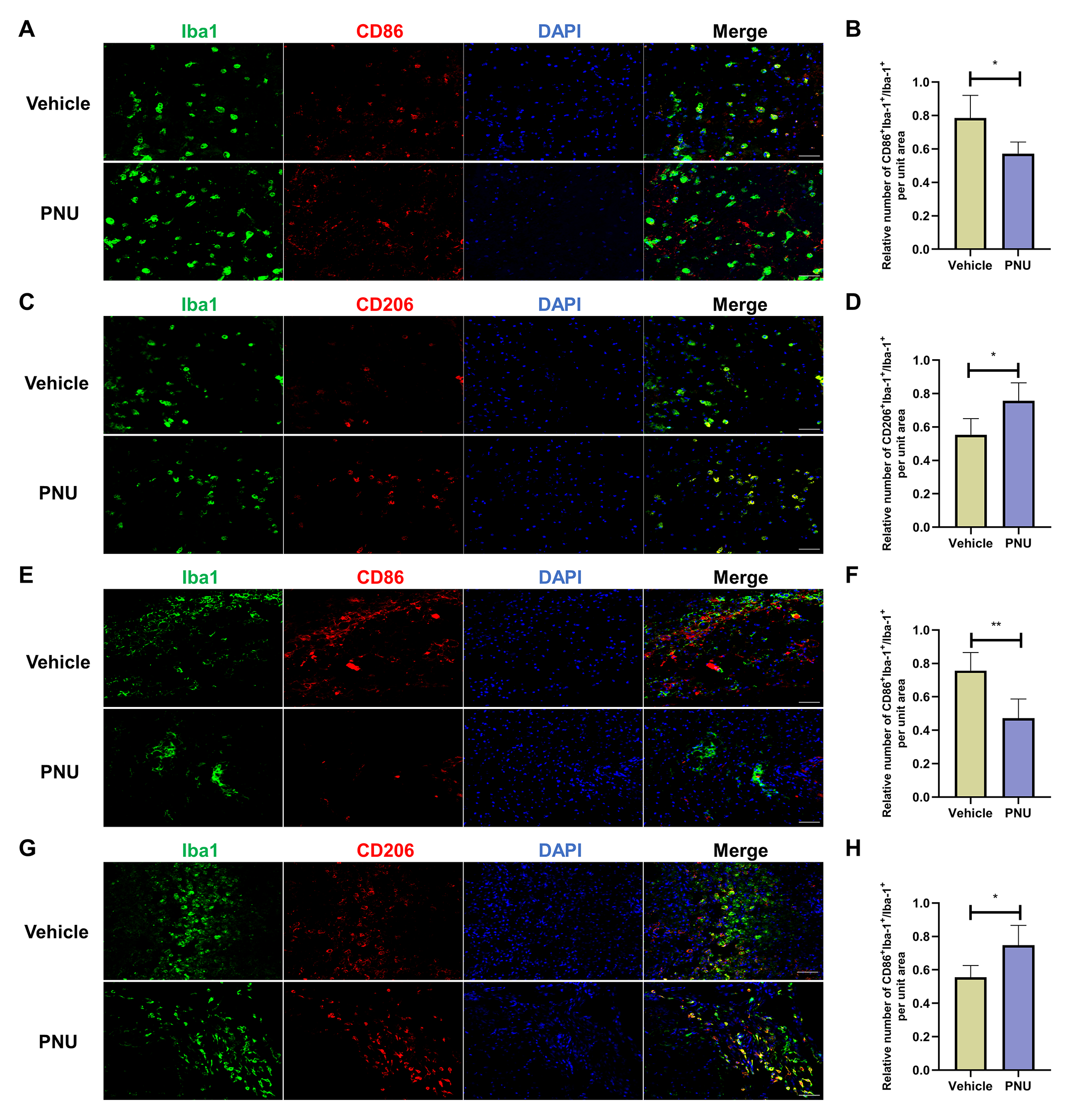

Supplement: Supplementary file 6 [file Image_5.TIF]
